# Environmental Epigenetics in Soil Ecosystems: Earthworms as Model Organisms

**DOI:** 10.3390/toxics10070406

**Published:** 2022-07-20

**Authors:** Maja Šrut

**Affiliations:** Department of Zoology, Center for Molecular Biosciences Innsbruck, University of Innsbruck, Technikerstraße 25, 6020 Innsbruck, Austria; maja.srut@uibk.ac.at

**Keywords:** earthworms, epigenetics, DNA methylation

## Abstract

One of the major emerging concerns within ecotoxicology is the effect of environmental pollutants on epigenetic changes, including DNA methylation, histone modifications, and non-coding RNAs. Epigenetic mechanisms regulate gene expression, meaning that the alterations of epigenetic marks can induce long-term physiological effects that can even be inherited across generations. Many invertebrate species have been used as models in environmental epigenetics, with a special focus on DNA methylation changes caused by environmental perturbations (e.g., pollution). Among soil organisms, earthworms are considered the most relevant sentinel organisms for anthropogenic stress assessment and are widely used as standard models in ecotoxicological testing of soil toxicity. In the last decade, several research groups have focused on assessing the impact of environmental stress on earthworm epigenetic mechanisms and tried to link these mechanisms to the physiological effects. The aim of this review is to give an overview and to critically examine the available literature covering this topic. The high level of earthworm genome methylation for an invertebrate species, responsiveness of epigenome to environmental stimuli, availability of molecular resources, and the possibility to study epigenetic inheritance make earthworms adequate models in environmental epigenomics. However, there are still many knowledge gaps that need to be filled in, before we can fully explore earthworms as models in this field. These include detailed characterization of the methylome using next-generation sequencing tools, exploration of multigenerational and transgenerational effects of pollutants, and information about other epigenetic mechanisms apart from DNA methylation. Moreover, the connection between epigenetic effects and phenotype has to be further explored.

## 1. Introduction

### 1.1. Epigenetics

The research field of epigenetics, directly translated as “above genetics” explores the non-genetic inheritance of variation in gene functions (phenotypes) occurring via mitosis or meiosis and describes the mechanisms behind this inheritance. Epigenetic phenomena are mediated through epigenetic marks which include DNA methylation, histone modifications, and non-coding RNAs [1]. These epigenetic marks shape the chromatin structure and are interconnected in a network that dictates the gene expression [2]. The best studied epigenetic mechanism is DNA methylation, which is a naturally occurring alteration that involves a methyl group addition to the cytosine (at the fifth carbon atom). This process involves the DNA methyltransferase (DNMT) enzyme which adds the methyl group (CH_3_) from S-adenosyl-L-methionine (SAM). The presence or absence of methyl groups can affect the coiling of DNA around histones and therefore the accessibility and binding of transcription factors [3]. DNA methylation most often occurs within C-G nucleotide pairs (CpG dinucleotides). This process is well-studied in vertebrates, which have highly methylated genomes with up to 70–80% of CpG methylation. On the other hand, invertebrate genomes are mostly sparsely methylated exhibiting a mosaic pattern of methylation with most of the methylation restricted to gene bodies (gene body methylation-gbM) and silenced repetitive elements [4]. The level of DNA methylation in invertebrates ranges from the complete absence or very low levels of DNA methylation in *Caenorhabditis elegans* and dipteran insects to high levels of methylation in some species of sponges [4,5,6].

DNA methylation can be detected with several methods offering insight into cytosine methylation at different resolutions. Methods can be divided into those profiling whole genome methylation and those focusing on the methylation status of specific genes of interest [7]. The gold standard for revealing the methylation of every cytosine in the genome is whole genome bisulfite sequencing (WGBS). This method is at the moment not so often used in environmental epigenetics due to the fact it bears high costs, and requires a sequenced genome and advanced bioinformatic skills. However methylomes of several invertebrate species have already been examined using WGBS and in few instances, the method has been applied in ecotoxicological context on *Drosophila melanogaster*, *Daphnia magna*, and *Crassostrea gigas* [8,9,10,11]. A good alternative to mitigate the costs and need for a reference genome and bioinformatic constraints of WGBS is to use the reduced representation bisulfite sequencing (RRBS) where only a fraction of the genome is sequenced. Methods such as EpiRADseq [12], epiGBS [13], and BsRADseq [14] provide cost-efficient high-throughput methylation data for many samples at the same time for only a fraction of the cost of WGBS. Furthermore, methods such as methylated CpG binding domain sequencing (MBD-seq) and methylation-dependent restriction site-associated DNA sequencing (mdRAD) proved to be reliable and cost-effective alternatives to WGBS as tested in corals [15].

DNA methylation/demethylation are mediated via DNMT and ten-eleven translocation (TET) enzymes. DNMT enzymes in animals include de-novo and maintenance DNMTs, where DNMT1 is described to maintain the DNA methylation during DNA replication whereas DNMT3 is vital for the establishment of genomic methylation patterns during development and is referred to as de-novo methyltransferase [4]. DNA demethylation can occur actively and is mediated via TET genes or passively by inhibition of DNMT1 activity. In earthworms, both DNMT1 and DNMT3 as well as TET genes have been found, however, their expression and activity could not be linked to altering DNA methylation patterns caused by exposure to pollutants [16,17].

The main role of DNA methylation in vertebrates is the silencing of inactive regions in the genome (transposable and viral elements). Unmethylated CpGs in vertebrates are mostly located in gene promoter regions within so-called CpG islands and are responsible for the regulation of gene transcription. In invertebrates, the prevalent type of CpG methylation is gbM [18]. GbM in invertebrates regulates transcriptional activity, alternative exon splicing, repression of intragenic promoter activity and reduces the efficiency of transcriptional elongation [19,20,21,22]. In invertebrates, gene bodies are either heavily or sparsely methylated which has been linked to high or low expression of genes, respectively [23]. It has been hypothesized that gbM could promote the predictable expression of essential genes for basic biological processes and modulate gene expression plasticity [19,24]. Furthermore, heavily methylated genes have been described as highly and broadly expressed, whereas lowly methylated genes are tissue-specific [23]. Across different invertebrate taxa, it was evident that genes with housekeeping functions, and constitutive and ubiquitous functions tend to be more methylated than those with inducible functions [24]. For example, in corals, weak methylation patterns increased the gene expression plasticity in response to environmental stress. It was speculated that the potential mechanism behind this phenomenon could be that weak methylation allows greater access to alternative transcription start sites, enabling in this way flexibility in gene expression plasticity [25]. This flexibility in gene expression was also confirmed in marbled crayfish, where gbM was found to be linked to stable gene expression and lowly methylated genes showed increased variation in expression levels [26]. Moreover, housekeeping genes of marbled crayfish that are unexpressed have low methylation of gene bodies, whereas those with moderate gene expression have higher levels of gbM [27]. Functional analyses in oysters revealed that the high expression level of genes correlates with high methylation [28,29]. Furthermore, hypermethylated genes in oysters are enriched for biological processes related to metabolism and housekeeping functions, whereas hypomethylated genes are associated with developmental processes, cellular communication, and adhesion [30].

### 1.2. Environmental Epigenetics

Environmental epigenetics studies the cause-effect relationships between various environmental factors such as nutrition, temperature, exposure to pollutants, etc., and the epigenetic modifications and changes of the organism phenotypes. These modifications can enable the adaptation of organisms to novel environmental conditions. However, in some cases, changes in epigenetic marks can lead to detrimental phenotypic endpoints, can persist for a long time, and even be heritable [31]. Since epigenetic marks respond to environmental pollution resulting in potentially heritable effects, those marks have been suggested as suitable candidates for the development of biomarkers of environmental exposure [31,32].

Changes in epigenetic marks, mainly DNA methylation, under various environmental stressors have been studied in different invertebrate taxa, including polychaetas, insects, corals, mollusks, and crustaceans, as summarized by several review articles [33,34,35]. Among these, the most data of high resolution obtained through WGBS are available for *D. magna*, corals and mollusks. In *D. magna* WGBS approach detected alterations in DNA methylation patterns in individuals exposed to gamma irradiation and the toxic cyanobacterium *Microcystis aeruginosa* [8,9]. In coral *Stylophora pistillata* exposed to low-pH environments, WGBS revealed changes in pathways regulating cell cycle and body size and suggested that DNA methylation enables fine-tuning of gene expression as a response to changing environmental conditions [36]. In pacific oysters *Crassostrea gigas* exposed to the herbicide diuron, an intergenerational epigenetic effect was revealed using a whole genome approach. Changes in DNA methylation patterns within coding sequences of unexposed mussels, coming from exposed genitors were revealed, indicating DNA methylation as an important pathway in phenotypic changes induced by environmental pollution [11].

As evident from these examples, although invertebrates have much lower levels of DNA methylation in comparison to vertebrates, they present promising models for environmental epigenetic studies. Additionally, their wide distribution, accessibility, easy laboratory maintenance, and limited ethical issues in comparison to vertebrate models, make invertebrate models even more attractive for environmental epigenetic studies. On the other hand, limited genomic data apart from commonly used model organisms, makes high-resolution methylation studies unavailable for many species. However, this is rapidly changing, as the sequencing costs are dropping and bioinformatics tools as well as genomic resources of many non-commonly used model organisms are becoming available.

### 1.3. Earthworms Role in Soil Ecosystems

Soil is a non-renewable resource essential for the well-being of the ecosystems, and its protection is one of today’s main challenges and priorities [37]. Soil acts as the major sink for pollutants released into the environment through anthropogenic activities, posing a severe risk for the ecosystem and ultimately for human health. To propose adequate measures for soil protection it is essential to understand the nature and the extent of pollutant effects on the health of the soil biota. Earthworms represent the majority of the animal biomass in the soil and are key invertebrate species, which perform an essential ecological role in the soil by improving its structure and fertility. By ingesting soil in the amount of 2 to 30 times their body weight per day, they exert a major impact on the physical, chemical, and microbiological properties of the soil and thus on the soil profile development [38]. Through their soil-dwelling activities, earthworms cause changes in nutrient availability, soil respiration, impact the composition and biomass of soil microorganisms, an abundance of other soil invertebrates, and composition of plant communities [39]. Due to their irreplaceable contribution to ecosystem goods and services, earthworms are often referred to as “ecosystem engineers”. Earthworms are found in different soil layers and are commonly classified into three ecological categories: (1) anecic species make permanent deep vertical burrows and feed in the litter from the soil surface; (2) epigeic species live on the soil surface and consume the litter; (3) endogeic species live in the soil making shallow burrows and feed on the organic matter within the soil.

## 2. Earthworms as Models for Environmental Stress Assessment

Earthworms represent one of the most relevant sentinel organisms for the assessment of soil pollution due to several advantages. Through soil dwelling activities they are in constant contact with the soil occurring both during soil ingestion and by passive absorption through the skin. They are present in a variety of soil horizons, abundant in contaminated soils and there are several standardized tests and molecular techniques on earthworms available [40]. Earthworms have frequently been applied in studies examining not only the effects of soil pollution but other anthropogenic stressors as well, by analyzing a suite of reliable and sensitive biomarkers at the molecular and cellular level [38,39]. Earthworms have also been found to exhibit remarkable tolerance to soils with high pollution burdens and were able to thrive and maintain abundant populations even in soils containing metal levels that significantly exceed toxic effect concentrations [41]. For instance, *Lumbricus rubellus* found in mining areas heavily polluted with arsenic and copper could survive arsenic-contaminated soil that was toxic for earthworms from clean sites [42]. This tolerance was ascribed to the genetic basis, as it was preserved in a culture over two generations [43]. Later on, this adaptive phenotype was suggested to also have an epigenetic background [44]. Therefore, both changes in the genetic sequence and epigenetic states contribute to adaptive phenotypes in earthworm species [41].

Earthworms as models in ecotoxicological research have already been recognized in the 1960s with the publication of the reports on negative effects of pesticides on several soil invertebrates [41,42]. The first international standardization of earthworm toxicity tests occurred through Organization for Economic Co-operation and Development (OECD) and focused on short-term (acute) responses such as survival [43]. This test turned the earthworm *Eisenia fetida* into the standard model organism for evaluating the effect of chemicals on terrestrial invertebrates. Further standardization tests also used this earthworm species to assess the effect of pollutants on different endpoints, including reproduction [40,44,45].

Due to the popularization through standardized tests, *E. fetida* is to this day one of the most commonly used earthworm sentinel organisms. Its main advantages include a short life cycle, ease of rearing in laboratory conditions, and representativeness among terrestrial fauna [46]. Apart from *Eisenia* species, also earthworms of the genus *Lumbricus* are considered one of the most relevant bioindicators for terrestrial ecosystem health monitoring, due to their irreplaceable roles in soil structure and fertility [47,48]. Native species of *Aporrectodea caliginosa* have also been proposed as relevant sentinel organisms for assessing the effects of anthropogenic pollution [49].

There has been a significant increase in the use of different earthworm species for toxicological tests and biomarkers, including measurements of changes at the molecular and biochemical level, determination of histological and behavioural changes and monitoring changes at the population and community levels. According to Spurgeon et al. [48], the biomarkers used on earthworms can be summarized in four main categories: (1) cellular tests that measure the effects of cellular function and integrity, such as neutral red retention assay, immune activity assays, comet assay; (2) measurements of detoxification enzymes and proteins activity, such as phase 1 cytochrome P450 enzymes, glutathione-S-transferase, superoxide dismutase, catalase, and peroxidase; (3) molecular responses to the effects of exposure, such as heat shock proteins, cytochrome C oxidase, lysosomal membrane linked genes, metal-containing enzymes; (4) effect on the transcriptome, proteome or metabolome. This last point could be supplemented by the effect on the epigenome, which has recently been recognized as an important part of the omics, particularly regarding its direct link to the expression of genes and its role in inheritance. The regulation and functionality of dynamic biological processes depend on interaction between different omics-genomics, epigenomics, transcriptomics, metabolomics, and phenomics [50]. Therefore, to truly understand fundamental biological processes, it is of paramount importance to understand separate omics and to disentangle their interactions [50]. In recent years, several research groups have recognized the importance of epigenome as an important predictor in evaluating the effects of environmental pollution on earthworms. So far, all studies focused solely on DNA methylation changes due to acute and chronic exposure to environmental chemicals, mostly metals.

## 3. Earthworms as Models in Epigenetic Research

Most commonly used earthworm models in environmental epigenetics are the species from the genus *Lumbricus*. In *L. rubellus* earthworms collected from sites across former arsenic (As) and copper (Cu) mines, an association of methylation patterns with soil arsenic concentrations in one earthworm lineage has been revealed [51]. The authors suggest that these earthworms could utilize epigenetic mechanisms to adapt and cope with the contamination. In a laboratory exposure experiment of *L. rubellus*, there were no methylation changes evident upon exposure to As and cadmium (Cd), however, fluoranthene was able to alter DNA methylation patterns evident using methylation-sensitive amplification polymorphism (MSAP) technique [52]. The same author examined also earthworm DNA methylation patterns in specimens collected from sites close to zinc (Zn), lead (Pb), and Cd smelter, however, no marked changes in DNA methylation were observed in comparison to earthworms from the control location [53]. In a series of experiments on *L. terrestris* exposed to environmentally relevant concentrations of Cd (10–25 mg/kg), DNA hypermethylation was recorded at several time points during 12-week exposure experiments [17,54]. However, these changes in DNA methylation could not be explained by common mechanisms involved in DNA methylation and demethylation, including the expression and activity of DNMT and TET genes [17]. Additionally, at the level of methalothionein gene (MT2), gene body methylation did not show any changes caused by exposure to low environmentally relevant Cd concentrations [17]. Moreover, the promoter region of the MT2 gene in *L. terrestris* does not possess any methylated cytosines [55]. To further explore the mechanisms between DNA methylation and the Cd detoxification process, *L. terrestris* earthworms were acutely exposed to high Cd concentration (200 mg) and demethylating agent (5-aza-20-deoxycytidine (Aza)) over the period of 48 h, however, no relationship was discovered [56].

Although commonly used for toxicological testing, earthworm species *E. fetida* was not often used for the assessment of epigenetic endpoints. Exposure of this earthworm species to serial concentrations of bisphenol A (BPA) for 28 days caused a decrease in DNMT1 and DNMT3b gene expression, indicating the BPA effect on the DNA methylation process [16]. Another earthworm species used for evaluation of DNA methylation included *Octolasion lacteum* exposed to ionizing radiation within the Chernobyl exclusion zone. DNA methylation profiles assessed using methylation-sensitive amplification polymorphism (MSAP) did not differ in comparison to the earthworms from clean locations [57]. In earthworms collected from gold and silver mines, the percentage of global DNA methylation was inversely correlated to total tissue concentrations of several metals [58]. The summary of these studies is presented in Table 1.

## 4. Advantages of Earthworm Models in Environmental Epigenetics

For environmental epigenomic studies earthworms offer several advantages over other invertebrate species:

- Environmentally relevant sentinel species. Earthworms are important and often used species in ecotoxicological research. A range of effects of acute and chronic exposure to pollutants both in laboratory settings as well as in situ have been described. Earthworms are key soil organisms, providing important ecosystem services such as improving soil fertility and structure. Therefore, they represent a relevant model organism to study the effects of soil pollution.
- High level of genome methylation for an invertebrate species. In earthworm species, *L. rubellus* and *A. caliginosa* around 13% of methylated cytosine content was determined [51,59]. Therefore, earthworm epigenome has an important role in adaptation to environmental challenges. For comparison, the commonly used invertebrate model *D. magna* has only around 0.85% of methylated cytosines [8].
- Epigenome is responsive to environmental stimuli. As reviewed in the last paragraph, earthworm methylome responds to exposure to environmental pollution and shows changes even in the case of low, environmentally relevant concentrations of pollutants.
- Availability of molecular resources. To assess the genome methylation at high resolution it is of paramount importance to have genomic data available. So far, the genome is sequenced in two earthworm species *E. fetida* [60] and *L. rubellus* (unpublished). Additionally, there is available transcriptome for *E. fetida* [61], *E. andrei* [62], and *L. terrestris* (unpublished).
- Possibility to study epigenetic inheritance. Generational studies in a laboratory setting can easily be performed on earthworms. For instance, the full generational time of *E. andrei* and *E. fetida* is between 63 and 83 days and they reach sexual maturity in 40 to 60 days allowing studies on longer-lived species in comparison to commonly used model organisms such as *D. pulex* (5–10 days generational time) and *C. elegans* (3–4 days generational time) [53].

**Figure 1:**
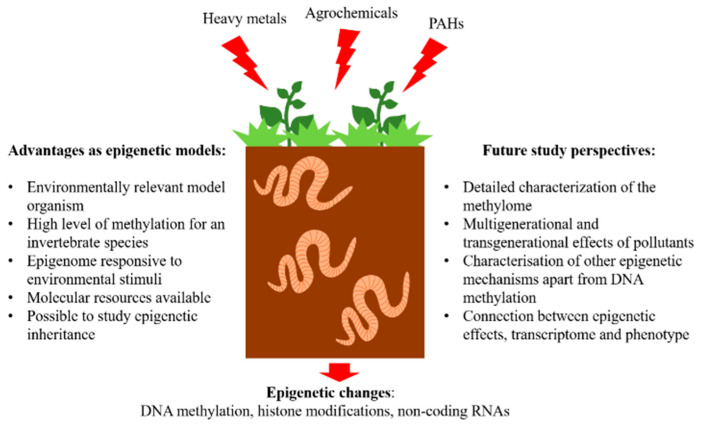
Earthworms as models in environmental epigenetics. Advantages and future study perspectives.

Earthworms present promising novel epigenetic models to study the impacts of soil pollution on the epigenetic landscape. Implementation of novel and non-standard models in epigenetic research offers ways to study various biological mechanisms underlying the natural diversity [63]. The use of earthworms as epigenetic models is still in its infancy and the studies so far have only scratched the surface in discovering the epigenetic landscape and its potential as a biomarker of past and present pollution burden in earthworms. Therefore, the obvious disadvantage of earthworms as epigenetic models is the lack of detailed genetic and epigenetic tools. This disadvantage presents at the same time a critical research challenge and lays the way for future study perspectives (Figure 1).

## 5. Future Study Perspectives

There are several knowledge gaps that should be examined to enable the widespread use of earthworms as models in environmental epigenetics:

- Detailed characterization of the DNA methylation landscape. All of the studies performed so far on earthworms used low-resolution methods such as MSAP. Although this technique might be a good approach to cost-effectively screen a large number of samples, it has a very limited resolution, as only small number of loci are screened. WGBS is recommended to obtain an overview of methylation in the entire genome, however, due to its high cost, the method can not be used on a large number of samples. To overcome this issue, it is possible to use reduced representation approach methods or targeted approaches, however, in this way risking the possibility to miss out on some important alterations. In any case, the use of the NGS approaches will significantly improve our understanding of the DNA methylation landscape in the earthworm genome and its perturbations when challenged with environmental pollution.
- Multigenerational and transgenerational effects of pollutants. Long-term exposures covering several generations of earthworms to environmental pollutants have been seldom performed. It has been observed that earthworms originating from contaminated sites tend to have lower reproductive fitness in laboratory settings and it is therefore difficult and time-consuming to perform experiments on F1 and F2 generations [64]. A long-term multigenerational and transgenerational study on earthworms *E. fetida* and *E. andrei* exposed to arsenic, cadmium, and imidacloprid was performed to assess the phenotypic effects caused by pollution on three exposed and two unexposed generations [53]. However, such long studies exploring the earthworm epigenome have so far not been performed. The assessment of multigenerational and transgenerational effects of pollutants is of paramount importance as it can inform on long-lasting impacts that can also be inheritable. For example, in an annelid worm *Enchytraeus crypticus* exposed to nanomaterials (CuO and nanostructured tungsten carbide cobalt (WCCo NMs)) over several generations, an increase in global DNA methylation was associated with phenotypic effects (reproduction) [65,66,67]. Additionally, the authors noticed transgenerational effects as well as reported both global and tissue-specific changes in DNA methylation patterns in F6 and F7 generations whose ancestors were exposed to Cu nanomaterials and Cu salt [65,67].
- Characterisation of other epigenetic mechanisms apart from DNA methylation. All epigenetic mechanisms including DNA methylation, histone modifications, and micro RNA are important to study topics. However, for earthworms, only DNA methylation has been, so far, studied in more detail.
- Connection between epigenetic effects, transcriptome, and phenotype. The connection between epigenetic changes, gene expression, and phenotypic endpoints such as growth, development, reproduction, etc., is of paramount importance as it can show adaptive responses triggered by epigenetic alterations. This connection is crucial when considering the inheritance potential of epigenetic changes and transgenerational effects. These kinds of studies present a critical research challenge and can aid in our understanding of the adaptability and plasticity potential of organisms under stressful environments.

## 6. Conclusions

Earthworms have been successfully used as models to study epigenetic alterations triggered by environmental pollution. For an invertebrate species, their genome is relatively highly methylated and the availability of molecular resources data, such as genome and transcriptome, for some earthworm species, makes it possible to use NGS approaches in the future. Furthermore, earthworm generational studies can be easily conducted in laboratory settings making them adequate models to study epigenetic inheritance. However, to adequately use earthworms as models in environmental epigenetics, there are still several knowledge gaps that need to be explored, such as the effects of pollutants on multigenerational and transgenerational epigenetic inheritance, the responsiveness of other epigenetic mechanisms apart from DNA methylation, the connection between epigenetic effects and phenotype as well as the use of NGS approaches for a detailed exploration of the earthworm methylome and its modifications in stressful conditions.

## Figures and Tables

**Table 1:** Summary of the studies evaluating the effects of environmental pollution on earthworms.

Toxicant	Model Earthworm	Epigenetic Endpoint	Method	Main Finding	Reference
Arsenic (As) and copper (Cu) mine	*L. rubellus*	Genome-wide DNA methylation	meAFLP *	Association of methylation patterns with soil As concentrations in one earthworm lineage	[51]
As, cadmium (Cd), fluoranthene	*L. rubellus*	Genome-wide DNA methylation	MSAP *	No effects of As and Cd, fluoranthene changed DNA methylation patterns	[52]
Zinc (Zn), lead (Pb), and Cd smelter	*L. rubellus*	Genome-wide DNA methylation	MSAP *	No methylation changes	[53]
Cd	*L. terrestris*	Genome-wide DNA methylation	MSAP *	Hypermethylation	[54]
Cd	*L. terrestris*	Global and gene-specific DNA methylation; *DNMT1*, *DNMT3*, *TET* gene expression and activity	Dot blot; bisulfite conversion and sequencing of the *MT2* gene body region; qPCR	Time and dose dependant changes in DNA methylation patterns; no significant changes in MT2 gene body methylation, no changes in DNMT and TET gene expression and activity	[17]
Cd	*L. terrestris*	Gene-specific DNA methylation	Bisulfite conversion and sequencing of the *MT2* promoter region	No methylation in the MT2 promoter region	[55]
Cd	*L. terrestris*	Global DNA methylation	Dot blot	No methylation changes	[56]
Bisphenol A (BPA)	*Eisenia fetida*	*DNMT1* and *DNMT3b* gene expression	qPCR	Lower expression at higher BPA concentrations	[16]
Ionizing radiation within the Chernobyl exclusion zone	*Octolasion lacteum*	Genome-wide DNA methylation	meAFLP *	No methylation changes	[57]
Silver and gold mine	Earthworms	Global DNA methylation	HPLC *	Inverse correlation between the percentage of methylated DNA and total tissue As, As + Hg, As + Hg + Se + Sb, and inorganic As + Hg	[58]

* meAFLP: methylation-sensitive amplified fragment length polymorphism; MSAP: methylation-sensitive amplification polymorphism; HPLC: high-pressure liquid chromatography.
